# Dynamic plant height QTL revealed in maize through remote sensing phenotyping using a high-throughput unmanned aerial vehicle (UAV)

**DOI:** 10.1038/s41598-019-39448-z

**Published:** 2019-03-05

**Authors:** Xiaqing Wang, Ruyang Zhang, Wei Song, Liang Han, Xiaolei Liu, Xuan Sun, Meijie Luo, Kuan Chen, Yunxia Zhang, Hao Yang, Guijun Yang, Yanxin Zhao, Jiuran Zhao

**Affiliations:** 1 Beijing Key Laboratory of Maize DNA Fingerprinting and Molecular Breeding, Maize Research Center, Beijing Academy of Agriculture & Forestry Sciences, Beijing, 100097 China; 2 Key Laboratory of Quantitative Remote Sensing in Agriculture of Ministry of Agriculture, Beijing Research Center for Information Technology in Agriculture, Beijing, 100097 China; 3 College of Architecture and Geomatics Engineering, Shanxi Datong University, Datong, 037009 China; 4 Key Laboratory of Agricultural Animal Genetics, Breeding and Reproduction, Ministry of Education, College of Animal Science and Technology, Huazhong Agricultural University, Wuhan, 430070 China

## Abstract

Plant height (PH) is a key factor in maize (*Zea mays* L.) yield, biomass, and plant architecture. We investigated the PH of diverse maize inbred lines (117 temperate lines, 135 tropical lines) at four growth stages using unmanned aerial vehicle high-throughput phenotypic platforms (UAV-HTPPs). We extracted PH data using an automated pipeline based on crop surface models and orthomosaic model. The correlation between UAV and manually measured PH data reached 0.95. Under temperate field conditions, temperate maize lines grew faster than tropical maize lines at early growth stages, but tropical lines grew faster at later growth stages and ultimately became taller than temperate lines. A genome-wide association study identified 68 unique quantitative trait loci (QTLs) for seven PH-related traits, and 35% of the QTLs coincided with those previously reported to control PH. Generally, different QTLs controlled PH at different growth stages, but eight QTLs simultaneously controlled PH and growth rate at multiple growth stages. Based on gene annotations and expression profiles, we identified candidate genes controlling PH. The PH data collected by the UAV-HTPPs were credible and the genetic mapping power was high. Therefore, UAV-HTPPs have great potential for use in studies on PH.

## Introduction

Maize (*Zea mays* L.) was domesticated from Balsas teosinte (*Zea mays* subspecies *parviglumis*) in southwestern Mexico around 9,000 years ago^1^. Subsequently, maize has been continuously improved by humans. The most important improvements spread from the tropical region to temperate regions, and can be viewed as adaptations^2^. The adaptation process allowed maize to be widely cultivated and to become the food crop with the largest production worldwide (http://faostat3.fao.org/compare/E). However, the world’s population is soaring and the demand for food is increasing. It has been reported that the world’s grain demand must increase by 70% by 2050^3^. Therefore, corn, the most widely cultivated grain, has become particularly important in safeguarding world food security.

Maize yield is highly complex and is affected by many factors, among which plant height (PH) is a particularly important factor because it affects not only lodging resistance, but also biomass and yield^4^. In the first Green Revolution, with the successful development of wheat and rice varieties with semi-dwarf genes (*rht1*, *sd1*), crop yields increased dramatically^5–7^. In fact, PH is so important that researchers have made unremitting efforts to explore its genetic mechanism. To date, a large number of quantitative trait loci (QTL) have been identified for maize PH using diverse genetic populations^8–13^. Some of these genes have been cloned, such as *an1*, *dwarf8*, and *br2*, and most encode proteins involved in the synthesis and transport of gibberellin and auxin^14–16^.

Maize PH shows different characteristics during the whole growth period, especially at the important growth stages, such as the seedling (V1–V5), jointing stage (V6–V10), flowering, and mature stages^17^. Usually, maize growth is slow at the seedling stage, fast at the jointing stage, gradually slows at the R stage, and stops at the milky maturity stage^18^. However, researchers have generally investigated PH at the mature stage to obtain the final height. This has led to a lack of systemic understanding of the entire PH development process and the genetic factors controlling it. The workload of manual measurement has also contributed to PH typically only being measured at the mature stage.

Manually measuring PH is a laborious and time-consuming task. Since plants are tall at maturity, errors in the measurement process are unavoidable and the accuracy of the data will be affected. In recent years, a series of high-throughput automated phenotypic systems have been developed, such as the Australian plant phenomics facility (https://www.plantphenomics.org.au/resources/). The concept of high-throughput phenotyping involves using autonomous tools with numerous sensors to automatically collect large amounts of data on plant variability across genetic lines^19^. At present, indoor platform systems in which environmental effects are minimized are widely used for dissecting phenotypic traits^20–22^. Compared with indoor platforms, the development of field high-throughput platforms requires high flexibility and a large payload, as the field environment is often complex^23–25^. High-throughput phenotypic platforms (HTPPs) for use in the field, especially those using remote sensing technology, can greatly facilitate the collection of crop trait data. Using unmanned aerial vehicles (UAV) for agricultural remote sensing is an innovative technology^19^. A UAV consists of a drone, its payload, and a ground control points (GCPs) for mission planning and flight control^19^. In recent years, UAV-HTPPs has been increasingly used in agricultural research, with advantages of manoeuvrability, suitability, high operational efficiency, non-invasiveness, and low cost. For example, UAV-HTPPs have been used to collect data on canopy reflectance, temperature, PH, and biomass of maize, wheat, and cotton^23,25,26^. Previous studies have estimated the PH of maize, barley, wheat, and sorghum in a field environment using various UAV-HTPPs^25,27–29^. With constant improvements in UAV equipment, the continuous optimization of image processing methods, and the removal of extreme data, the correlation coefficients between PH measured by UAV remote sensing (PH_UAV_) and PH measured with a ruler (PH_R_) have reached moderate to high values^25,27–29^.

To better understand the dynamic PH mechanism, we investigated PH at four important growth periods with a UAV system for genetically diverse maize inbred lines that are widely used in maize genetic research^9,13,30^. Through this design, we aimed to explore more PH characteristics with the aid of the high-throughput UAV and data processing procedures, and then dissect the genetic basis of PH for different maize groups at different stages.

## Results

### High-throughput Digital PH Extraction and Validation

**Figure 1:**
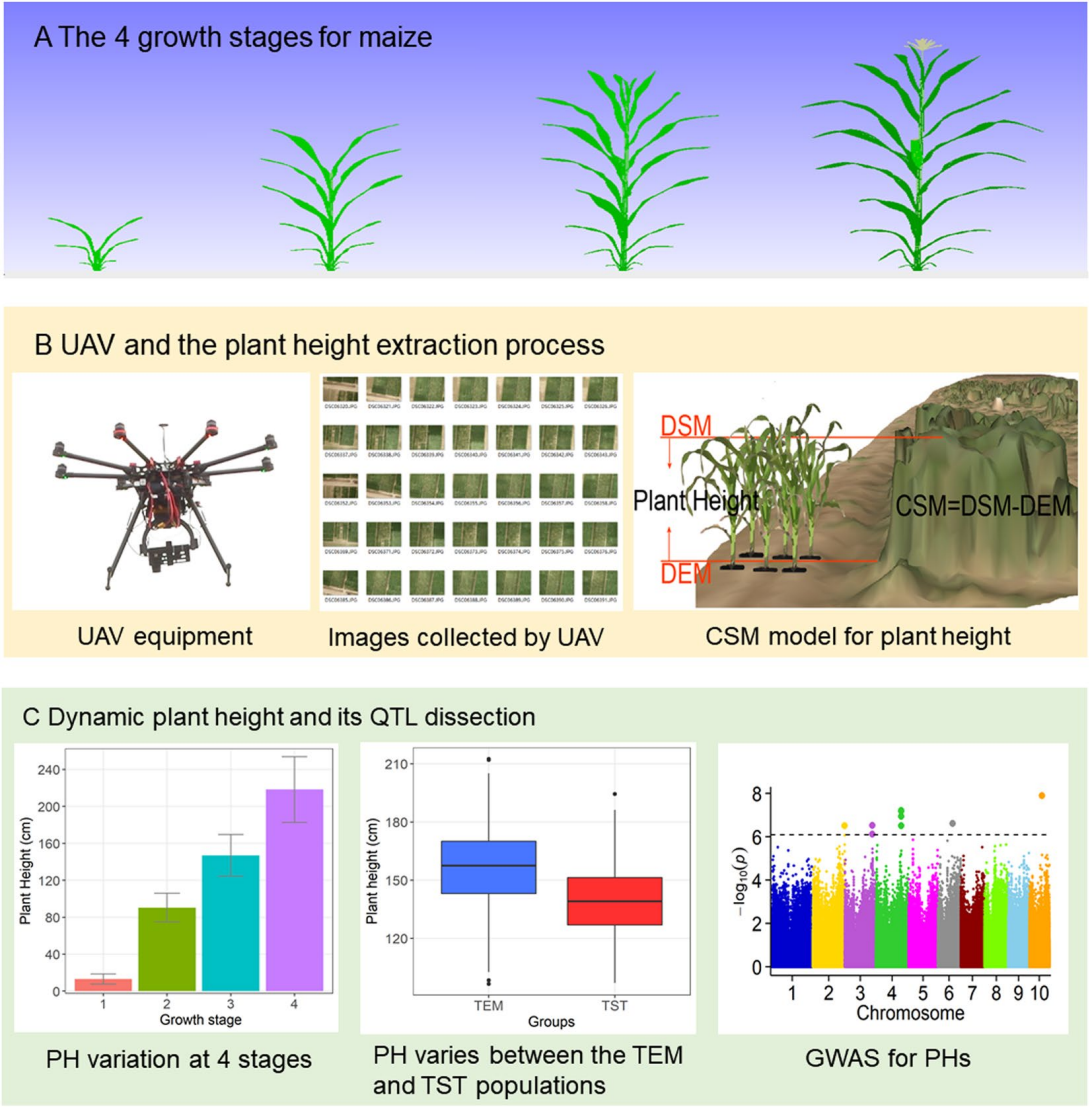
Field high-throughput phenotyping for plant height. (**A**) Digitally designed graphs depicting maize plants at four growth stages. Graphs were constructed using PlantCAD-maize software. (**B**) Unmanned aerial vehicle equipment and plant height extraction process. (**C**) Dynamic plant height and quantitative trait loci (QTL) dissection. Whole procedure included trait variation analysis and genome-wide association study.

To investigate the PH of 117 temperate and 135 tropical maize inbred lines at four growth stages, we used the UAV-HTPPs to collect image data. Four flights were conducted during the whole growth period of maize; one flight at each of the V5, V12, V15, and R stages (at 24, 45, 57 and 80 days after sowing, respectively; Fig. 1A; Table 1). On each flight, the average flight altitude was 52.5 m. A total of 559 original images were taken on the four flights.

**Table 1:** The investigation date for plant height using UAV.

Flight	Date	DAS^a^	DAFD^b^	Development stage	Description
1	8 June 2017	24	—	V5	seeding stage
2	29 June 2017	45	21	V12	jointing stage
3	11 July 2017	57	12	V15	trumpet
4	3 August 2017	80	23	R	flowering stage

Note: ^a^DAS means days after sowing.

^b^DAFD means days after the closest former date.

Using an automated data extraction process developed by our group, we first filtered the original images, and retained 460 high-quality images (average, 115 images per flight). Agisoft PhotoScan (full-featured trial version 1.3, Agisoft LLC, St. Petersburg, Russia) software was used to stitch digital images and reconstruct digital surface models (DSMs) and orthomosaics. This process mainly included the georeferenced import, GCPs import, image alignment, building a dense point cloud and building DSM. After reconstructing the orthomosaic model, the obtained image ground resolution was 1.13 cm/pixel (Table 2). The average image accuracy of the DSM was 2.31 cm/pixel. The digital elevation model (DEM) was generated by using Kriging spatial interpolation of the DSM from 1332 points located on the surface of the bare land using ArcMap (version 10.2, Esri Inc., Redlands, California, USA). Finally, we obtained the crop surface models (CSM) containing bare soil (DSM − DEM, Fig. 1B), which is equivalent to crop height. The mean crop height ranged from 9.6 to 253.4 cm among the four growth periods, and the average growth rate was 4.06 cm/d (Fig. 1C; Table 3).

**Table 2:** Evaluation of the accuracy of DSMs and orthomosaics.

Flight	Altitude (m)	Orthomosaic Resolution (cm/pix)	DSM Resolution (cm/pix)	Total error (cm)	Point Density (points/cm²)
1	40	0.72	1.44	1.46	47.9
2	60	1.33	2.45	2.17	14.2
3	60	1.35	2.47	2.08	13.7
4	60	1.11	2.12	5.75	20.2

**Table 3:** Statistic analysis for 7 plant height related traits for the BOTH group during 4 growth stages.

Trait	Max (cm)	Min (cm)	Mean (cm)	Sd (cm)	CV (%)
PH_1	31.34	5.35	13.66	5.07	37.09
PH_2	149.99	54.28	90.42	15.46	17.1
PH_3	212.56	96.52	146.98	22.63	15.4
PH_4	325.01	112.9	218.26	35.58	16.3
GRPH_1t2	16.05	2.26	6.47	2.7	41.75
GRPH_2t3	1.32	0.12	0.64	0.18	28.47
GRPH_3t4	1.27	0	0.51	0.25	49.61

**Figure 2:**
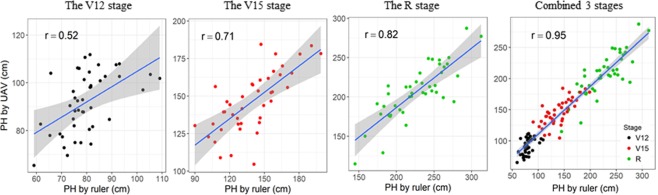
Linear relationship between plant height estimated from unmanned aerial vehicle (UAV) data and that measured manually at three growth stages. Blue solid line shows regression line, grey shadow represents 99% confidence interval.

To verify the accuracy of the PH data extracted using UAV-HTPP (PH_UAV_), 44 plots were randomly selected for PH measurement by ruler (PH_R_) at the same time at the second, third, and fourth flights. Linear regression models between PH_UAV_ and PH_R_ were established for each stage and for the three stages combined (Fig. 2). At the time of the second, third, and fourth flights, the correlations (*r*) were 0.52, 0.71, and 0.81, respectively, indicating that the accuracy of the PH_UAV_ data increased with the growth of maize plants. In addition, when data for the three stages were combined, the correlation coefficient was much higher (*r* = 0.95).

### Plant Height Varies Greatly Among Different Stages of Development

Based on the accurate PH data obtained by UAV-HTPP, we further analysed the variation in maize PH across the four growth stages among the three different groups (Table 3). For the combined temperate and tropical maize group (BOTH), the average PH was between 13.66 and 218.26 cm from the first to the fourth flight. Although the detected PH constantly increased during the four stages, the growth rate of plant height (GRPH) was highest in the first interval from the first to the second flight (1t2), and slower rate of growth in the time intervals from the second to the third flight (2t3) and the third to fourth flight (3t4).

**Figure 3:**
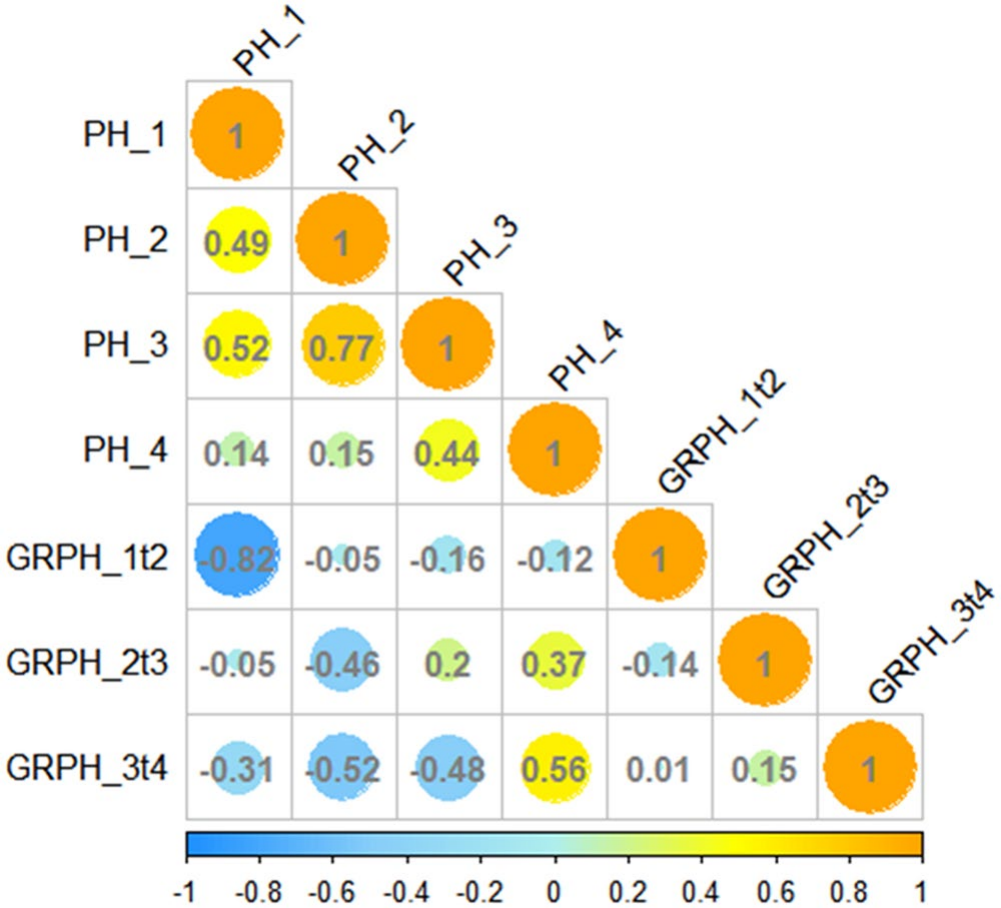
Correlation coefficient matrix among seven plant height-related traits. Yellow and blue indicate positive and negative correlations, respectively. Size of circle is proportional to correlation coefficient (number).

We conducted correlation analyses for the BOTH group to reveal the relationships among the seven PH traits (Fig. 3). The PHs at different stages were strongly positively correlated (*r* = 0.14 to 0.77), with the highest correlation coefficients for PH_2 and PH_3. The correlations between GRPH and PH were mainly negative, except for correlations between PH_4 and GRPH_2t3 and GRPH_3t4, and between PH_3 and GRPH_2t3. The correlations among GRPH at various stages were very weak.

**Figure 4:**
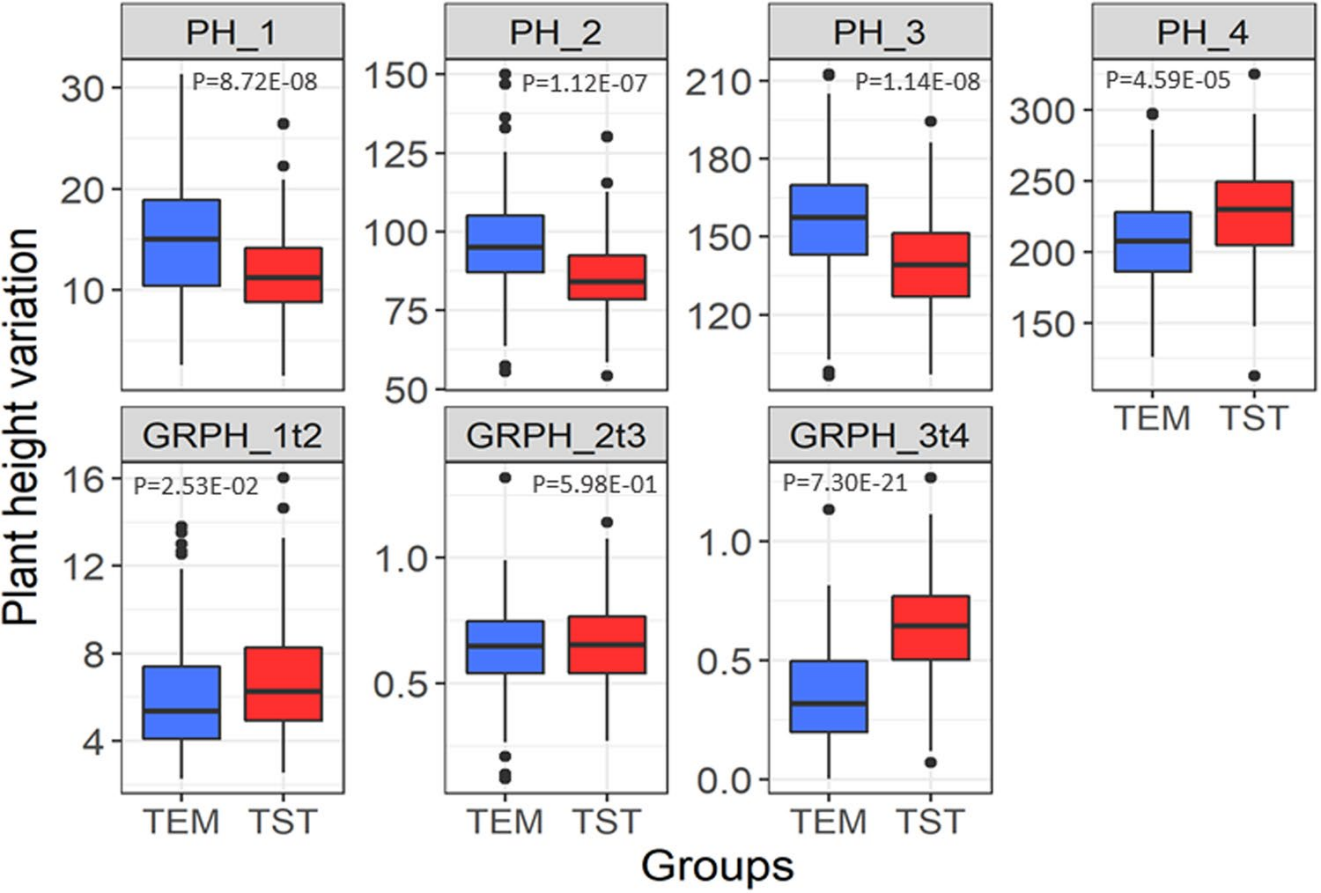
Plant height and related trait variations between temperate (TEM) and tropical (TST) populations at four growth stages. Blue and red box represent TEM and TST populations, respectively. Line in box plots shows median value. Box edges represent first and third quartiles, and dots outside whiskers represent value over 1.5 × interquartile range. Stars indicate that phenotypic distribution is significantly different (*P* < 0.05).

The BOTH group was very diverse, so we divided it into the temperate maize group (TEM) and the tropical maize group (TST). The PH differed significantly between TEM and TST at each growth stage (Fig. 4). From the first to the third stages, the PH was significantly greater for TEM than for TST. However, at the fourth stage, the PH of TST was greater than that of TEM. The trends were different for GRPH. As shown in Fig. 4, the growth rate of TST was significantly higher than that of TEM in GRPH_1t2 and GRPH_3t4, but not significantly different in GRPH_2t3.

On the basis of the differences in PH and GRPH between TEM and TST, we were able to describe the growth characteristics of the two groups. The TST lines grew slowly after emergence, resulting in shorter PH_1 for TST than for TEM in the first stage. From the V5 to the V12 stage, TST lines grew rapidly, resulting in higher GRPH_1t2 for TST than for TEM. However, because the base PH was much lower for TST than for TEM, the PH_2 was still lower for TST than for TEM. Because of their greater PH, TEM entered the V12 stage earlier than TST, and the period from the V12 stage to the tasselling period was the fastest growing period. Therefore, at the 2t3 stage, there was no significant difference between TEM and TST in GRPH_2t3. However, when TEM reached the flowering stage, the growth rate stabilized, while TST did not fully enter the flowering stage due to the photoperiod. Consequently, GRPH_3t4 and PH_4 were higher for TST than for TEM.

### Genetic Basis Affecting the Dynamic Development of Plant Height

**Figure 5:**
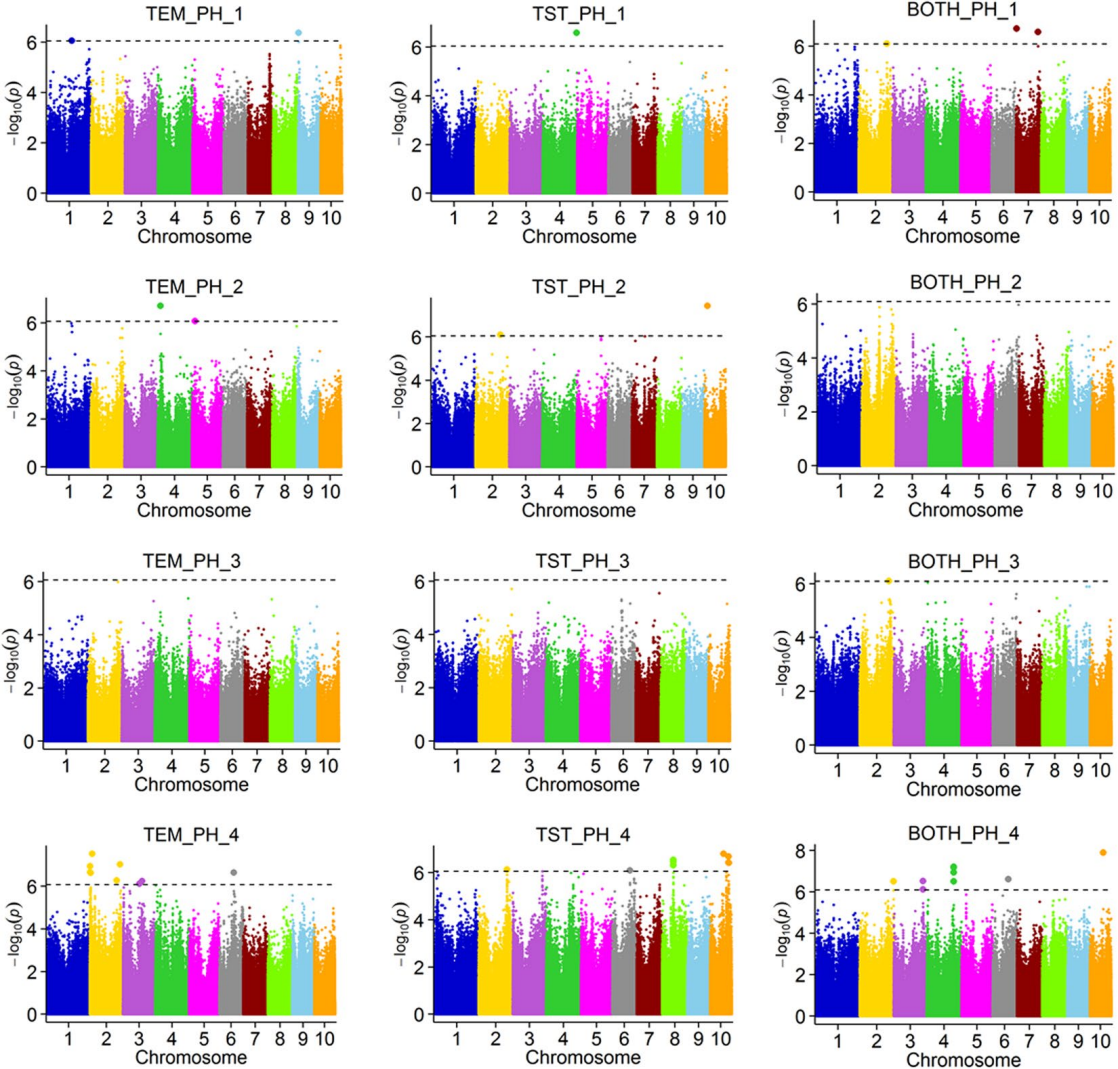
Genome-wide association study for plant height at four stages among temperate (TEM), tropical (TST), and both (BOTH) maize groups. Different colours represent different chromosomes. Dotted line is threshold. Single nucleotide polymorphisms (SNPs) above the threshold showed significant association.

**Figure 6:**
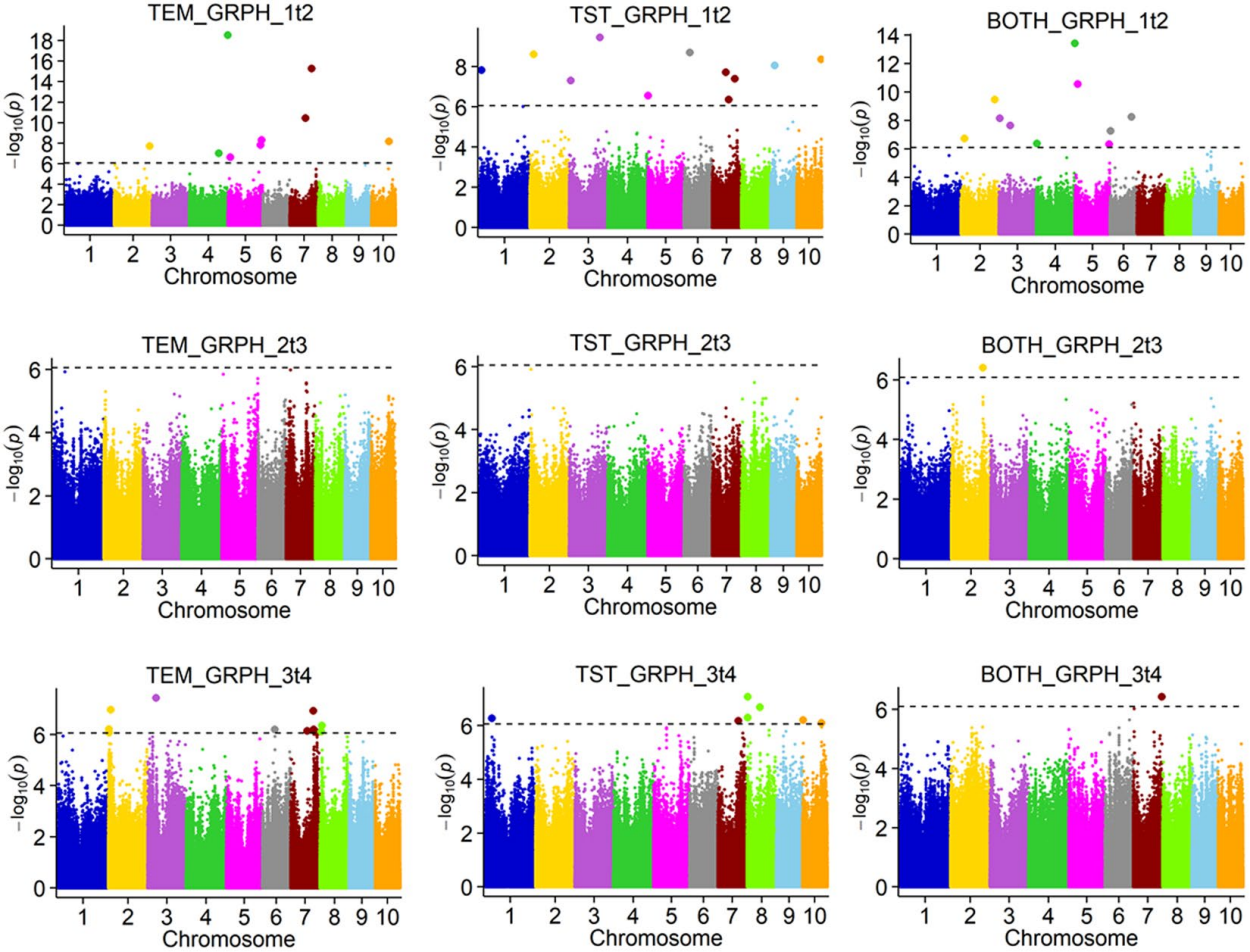
Genome-wide association study for growth rate at three time intervals among temperate (TEM), tropical (TST), and both (BOTH) maize groups. Different colours represent different chromosomes. Dotted line is the threshold. Single nucleotide polymorphisms (SNPs) above threshold showed significant association.

In view of the above-mentioned differences in PH and related traits among maize groups and different growth stages, we conducted a genome-wide association study (GWAS) for the seven PH-related traits in the TEM, TST, and BOTH groups (Figs 5–6; Supplementary Figs S1–S2). A total of 88 QTLs were detected, covering 10 chromosomes of the maize genome (Supplementary Tables S1–S2). We detected 38 QTLs for PH traits and 50 QTLs for GRPH traits among the three groups.

The physical locations of some QTLs overlapped. Therefore, we merged the overlapped QTLs or those whose physical distances were separated by less than 1 M but been identified as one QTL in other studies. Finally, we obtained 68 unique QTLs (Supplementary Table S3).

To verify the accuracy of the QTLs, we compared them with previously reported QTLs and genes related to PH. We found that 24 QTLs (35%) had been identified in previous studies (Supplementary Table S3)^8–13^. Genes involved in the auxin pathway, such as *ARFTF4* and *SAUR71*, were also found to be associated with PH^9^. Furthermore, 65% of QTLs were newly identified in our study, including QTLs for traits related to PH and GRPH. The characteristics of PH-related QTL were as follows.

First, the PH at different growth stages was mainly controlled by different QTLs, and a few QTLs controlled PH at multiple growth stages. We detected 6, 6, 2, and 24 loci for PH traits at the V5, V12, V15, and R stages, respectively (Fig. 5; Supplementary Table S1). More QTLs for PH were detected at the flowering stage than at other stages. Based on gene expression profiles and annotations, we identified some candidate genes for PH (Supplementary Table S3). For example, at the R stage, a strong candidate gene was *ARFTF4*^9^, which was detected in the QTL located at chr2:2.49–4.36 Mb in the TEM group. This gene was likely related to the control of PH, especially at the later stages of development. We also detected a candidate gene *SAUR71* at the early growth stage (PH_1) in the TST group. This gene was highly expressed in the internode of the maize inbred line B73.

We identified eight QTLs that simultaneously controlled PH and GRPH at different developmental stages (Supplementary Table S3). For example, one QTL located on chr2: 196.90–197.83 Mb was simultaneously detected for PH_1 and PH_4 traits, as reported in a previous study^11^. This QTL contained 32 genes, including the candidate gene *SAUR61*, which encodes an auxin response protein. This was identified as the most likely candidate gene because of the function of auxin in PH and its expression in the B73 internode. Another QTL located on chr4: 3.65–5.81 Mb was simultaneously detected for GRPH_1t2, PH_3, and PH_4, and was also reported previously^11^.

Second, there were 31, 1 and 18 QTLs detected for growth rate at stages at 1t2, 2t3 and 3t4, respectively, among the three groups (Fig. 6; Supplementary Table S2). QTL number was the least at the stage of 2t3. At the other stages, all the three groups have detected QTLs. Furthermore, we have also proposed three candidate genes in these QTLs, including two auxin-related genes and one encoding a growth-regulating factor 9-like (GRF9) protein (Supplementary Table S3). Among the eight overlapping QTLs, six controlled both GRPH and PH. For example, the QTL located on chr2: 191.56–192.53 Mb was simultaneously detected for GRPH_2t3 and PH_4. This QTL contained 15 genes, including the candidate gene encoding the gene auxin efflux carrier pin11. At the late development stage, two QTLs related to PH and GRPH were identified. One QTL was located on chr2: 2.49–4.36 Mb, and harboured the candidate gene *ARFTF4*. This QTL was detected for GRPH_3t4 in the TEM group, indicating that PH surveyed at the fourth stage was affected by the growth rate at the 3t4 stage. Another QTL was located on chr2: 16.12–17.89 Mb. The candidate gene in this region encoded the auxin-independent growth promoter, GRMZM2G142664, and showed high expression in the B73 internode.

## Discussion

Recently developed genomics-assisted breeding methods with novel technologies include next-generation sequencing, GWAS, and genomic selection. However, phenotyping is still time consuming. Genetic improvement is the most effective way to increase crop yields. With the rapid development of sequencing technology, genomic research has greatly increased, but phenotyping is the bottleneck in genetic research and breeding^24,30^. Consequently, there has been rapid development of HTPPs to obtain data for more traits for the rapid development of genetics and breeding.

A series of indoor phenotypic platforms have been developed for use in genetic studies^21,22,31^. For example, Yang *et al*. (2014) developed a high-throughput rice phenotyping facility (HRPF) to monitor 13 traditional agronomic traits, and defined two new traits based on data collected in this system. This resulted in the identification of 141 association loci, demonstrating that HRPF has the potential to replace traditional phenotyping techniques and can provide valuable gene identification information^21^. Zhang *et al*. (2017) monitored 106 traits in a maize recombinant inbred population across 16 developmental stages using an automatic indoor phenotyping platform, and identified 988 QTLs^22^. The application of these high-throughput, automated phenotyping systems can greatly shorten the phenotypic investigation time, ensure the accuracy of the phenotype, and allow for the discovery of phenotypes that could not be discovered using conventional techniques. More importantly, the traits discovered using the high-throughput platform can identify known genes as well as new loci, providing increased capacity for gene identification.

Compared with indoor platforms, field HTPPs are much more complex because they must be highly flexible and able to accommodate a large payload^24^. To date, UAV have proved to be excellent tools for high-throughput data collection in the field, and have achieved great success in studies on wheat, sorghum, and cotton^25,27–29^. In a study on PH in sorghum, UAV-HTPPs carried either a RGB or near-infrared, green, and blue (NIR-GB) camera. The NIR-GB camera and the 50th percentile of the DSM served as indicators of height. However, the correlation coefficient between PH_UAV_ and PH_R_ was only 0.523, leaving much room for improvement^29^. Holman *et al*. (2016) developed a method to investigate the PH of wheat, and the correlation between PH_UAV_ and PH_R_ was >0.9^25^. In this study, remote sensing-based UAV-HTPPs were used to extract PH data for maize at four different growth stages. The overall correlation between PH_UAV_ and PH_R_ was 0.95, which is higher than that of sorghum but a little lower than that of wheat^25^. However, the correlation between PH_UAV_ and PH_R_ was lower at individual stages (*r* = 0.52, 0.71, 0.81 for V12, V15, and R, respectively), than over the whole growth period.

As maize plants grew and their PH increased, the correlation between PH_UAV_ and PH_R_ increased gradually. This may be because the plant density increased to form a continuous canopy. Since most of the matching points are captured from the canopy, the DSM would more accurately reflect PH as the canopy became denser. Due to the lower planting density of maize than wheat or barley, maize plants are sparse and short in the growth early stages. Consequently, the matching points not only included the top of the canopy but also the ground, leading to errors in DSM in PH measurements^28,29^. In the future, the accuracy of UAV remote sensing data can be improved by improving equipment accuracy, improving image processing technology, and/or increasing the number of plants measured. More accurate data will benefit crop breeding programs.

To detect variations in PH, an in-depth investigation of phenotype is required. Currently, most studies determine PH at the mature stage, which can obtain data for the stable trait, but useful PH information is likely to be missed. In this study, we divided the maize growth period into four stages, and surveyed PH from the seedling stage to the flowering stage. We found that the GRPH of maize varied greatly among different stages of development. The growth rate was fastest at the 1t2 stage (GRPH = 6.47) and slowest at the 3t4 stage (GRPH = 0.51). We also found that TST maize grew slower and reached a shorter PH than did TEM maize from sowing to the V12 stage. For example, at the V5 stage, the PH for TST and TEM was 12.04 cm and 15.48 cm, respectively. At the V12 stage, the PH was still lower in TST than in TEM (139.64 cm *vs*. 155.5 cm). However, from V12 to the R stage, TST had a faster growth rate, resulting in taller plants than TEM at maturity (GRPH_(TST *vs*. TEM)_ = 0.63 *vs*. 0.36; PH _(TST *vs*. TEM)_ = 226.63 cm *vs*. 208.52 cm).

A large number of QTLs were detected using a GWAS for PH and GRPH. Of those, 35% had been reported previously. We detected 6, 6, 2, and 24 QTLs for PH traits at the V5, V12, V15, and R stages, respectively. Previous studies have used the same maize population to detect QTLs affecting PH, and the PH was measured manually using a ruler at the R stage^9,13^. We detected many more QTLs in this study, where we studied four periods, than in previous studies focusing on only one period. Some QTLs were detected in this study and in previous studies (Supplementary Table S3).

We found that different genes regulated PH at different stages. Some genes controlled PH at the early growth stage, such as *SAUR71*, which was detected in the TST group for PH_1^9^. Some genes controlled PH at later stages, such as *ARFTF4*, which was detected in the TEM group for GRPH_3t4 and PH_4^9^. We identified many QTLs related to PH and GRPH, and only a few overlapped. These results were consistent with those of Yan^32^, who investigated PH in five periods and found that QTLs controlling PH were expressed differently in different periods. The above results indicate that, if we assess PH over different growth stages, we will be able to identify more genes affecting this trait. We also identified eight overlapping QTLs that simultaneously controlled PH and GRPH at different stages. For example, the QTL located on chr2: 196.90–197.83 Mb controlled PH1 and PH4, and the same QTL was detected in a previous study on PH at the R stage^11^. Another QTL located on chr7: 136.64–140.92 Mb controlled GRPH_1t2 and GRPH_3t4, and was previously detected as a QTL for PH at the R stage^12^. The dynamic phenotype enables us to have a clearer understanding of plant developmental processes. The use of dynamic phenotypic data for mapping can identify more QTLs affecting the development of the PH trait. This is of great importance for the analysis of the genetic basis of traits and subsequent improvement of those traits.

## Materials and Methods

### Plant Materials and Experiment Design

The maize natural population used in this study was a subset of that described by Yang^33^, and consisted of 117 temperate lines and 135 tropical lines. The population had a high-density genotype of 1.25 million single nucleotide polymorphism (SNPs) with minor allele frequency (MAF) of >0.05^34^. The population was sown on 15 May 2017 at Xiao Tang Shan, Changping, Beijing National Precision Agriculture Research Center, Beijing, China (119.39°E, 40.17°N). The land plots were flat, with uniform soil fertility. Each row was 2 m long, and contained eight plants. Each plot consisted of three rows, with 65 cm distance between rows. Phenotypic data were collected by the UAV on days with clear sky and no wind (8 June, 29 June, 11 July, and 3 August 2017) (Table 1; Fig. 1A). On the same days, the height of plants in 44 randomly selected plots was manually measured with a ruler.

### Plant Height Image Data Extraction and Verification

The drones and ground monitoring platforms have been described in detail by Han *et al*.^35^. The key features can be summarized as follows: (1) An Octocopter UAV (DJI Spreading Wings S1000) platform integrating a 20.2-megapixel digital camera (Cyber-shot DSC-QX100) was used to collect a set of aerial images across four flights (Fig. 1B). (2) In each flight, the same flight plan was followed with 80% forward overlap and 75% side overlap at an altitude of approximately 40–60 m. (3) International standards organization sensitivity and shutter speed were set to automatic, and the focal length was fixed at 10.4 mm. (4) The flight time was within 15 min. (5) Sixteen GPS distributed evenly within the field were used to obtain accurate geographical references and improve the accuracy of plant-height extraction. The GCPs were located with millimetre accuracy by using a differential global positioning system (DGPS, South Surveying & Mapping Instrument Co., Ltd., Guangzhou, China).

The PH data extraction process has been described in detail by Han *et al*.^35^. The entire process can be summarized as follows: (1) The DEM was interpolated (Kriging spatial interpolation) from the first DSM from 1332 points that were not covered with vegetation by using ArcMap (version 10.2, Esri Inc., Redlands, California, USA). (2) The CSMs were calculated by subtracting the DSM at different plant growth stages from the DEM^36–38^ (Fig. 1B). (3) Plants were differentiated from soil using the excess green index proposed by Woebbecke^39^. (4) The PH was obtained by analysing CSMs using the Kriging spatial interpolation and maximum adjacent pixel methods to produce the optimal representative value. (5) The accuracy of PH estimates from UAV data was assessed by comparing estimated values with measured PH values by ruler in 44 randomly selected plots at the second, third, and fourth stages of plant growth. A linear regression model was applied across multiple dates using R v. 3.2.4 statistical software.

### Plant Height Variation between Temperate and Tropical Maize Lines

A total of 252 maize inbred lines, consisting of 117 temperate lines and 135 tropical lines, were used in this study. The PH was evaluated at four different growth stages, and seven PH-related traits were calculated: four absolute PH traits and three GRPH. The PH represents the absolute PH at each time point. The GRPH was calculated as the increase in PH divided by the initial PH, for example, GRPH_1t2 is the increase in PH from PH_1 to PH_2, divided by PH_1. The phenotypic distribution was analysed and graphs were constructed using R v. 3.2.4 statistical software.

### Association Analysis for Plant Height

A GWAS was carried out for the TEM, TST, and BOTH populations. Genotype data quality control with MAF >0.05 was performed separately for each population, with 1,141,328, 1,110,483 and 1,227,441 SNPs remaining for the TEM, TST, and BOTH groups, respectively. We used seven PH-related traits in the GWAS program, including all the PH and GRPH traits for the three groups. Combined with phenotypes and genotypes, the FarmCPU model in the MVP software package, which iteratively uses fixed and random effect models, was used for association tests in the TEM and TST groups with only kinship considered^20,40^. For the BOTH population, the top five principal components were added into the FarmCPU model to control false positives, which may be caused by population stratification and non-genetic effects^20,40^. The adjusted Bonferroni method (i.e., *P* ≤ 1/N, where N is the total number of genome-wide SNPs) was used as the global P value cut-off to declare significance of SNPs associated with a given trait. The *P* values were 8.76e^−7^, 9.0e^−7^, and 8.14e^−7^ for the TEM, TST, and BOTH populations, respectively. The QTL intervals were calculated as the upstream and downstream 100 kb for each significant SNP^41^. In each QTL interval, the SNP with the lowest *P* value was considered as the peak SNP. The QTL whose physical positions overlapped, and those that were less than 1 M apart and had been identified as one QTL by other researchers were considered as overlapping QTLs.

We searched for genes in each QTL according to the physical position of each gene in maizeGDB (https://www.maizegdb.org/). Gene annotations were based on maizeGDB, the InterProScan database (http: //www. ebi.ac.uk/interpro/scan.html), and the Gramene database (http://www.gramene.org/). Candidate genes were nominated based on two criteria; first, they should be functional relevance, and second, they should be expressed in the internode of maize inbred line B73 which had the reference genome in maizeGDB.

## Supplementary information

Supplementary Files
