# Supplementary material for: Dynamic plant height QTL revealed in maize through remote sensing phenotyping using a high-throughput unmanned aerial vehicle (UAV)
Source: Sci Rep. 2019 Mar 5;9:3458. doi: 10.1038/s41598-019-39448-z (PMC6401315; doi:10.1038/s41598-019-39448-z)
Supplement: Supplementary file 1 — Supplementary Files [file 41598_2019_39448_MOESM1_ESM.pdf]

SUPPORTING INFORMATION

Dynamic plant height QTL revealed in maize through remote sensing phenotyping using a high-throughput unmanned aerial vehicle (UAV)

Xiaqing Wang[1], Ruyang Zhang[1], Wei Song[1], Liang Han[2,3], Xiaolei Liu[4], Xuan Sun[1], Meijie Luo[1], Kuan Chen[1], Yunxia Zhang[1], Hao Yang[2], Guijun Yang[2], Yanxin Zhao[1,*], Jiuran Zhao[1,*]

[1]Beijing Key Laboratory of Maize DNA Fingerprinting and Molecular Breeding, Maize Research Center, Beijing Academy of Agriculture & Forestry Sciences, Beijing 100097, China

[2]Key Laboratory of Quantitative Remote Sensing in Agriculture of Ministry of Agriculture, Beijing Research Center for Information Technology in Agriculture, Beijing 100097, China.

[3]College of Architecture and Geomatics Engineering, Shanxi Datong University, Datong 037009, China.

[4]Key Laboratory of Agricultural Animal Genetics, Breeding and Reproduction, Ministry of Education, College of Animal Science and Technology, Huazhong Agricultural University, Wuhan 430070, China .

Xiaqing Wang and Ruyang Zhang contributed equally.

[*] Correspondence:

Yanxin Zhao, Email: rentlang2003@163.com

Jiuran Zhao, Email: maizezhao@126.com

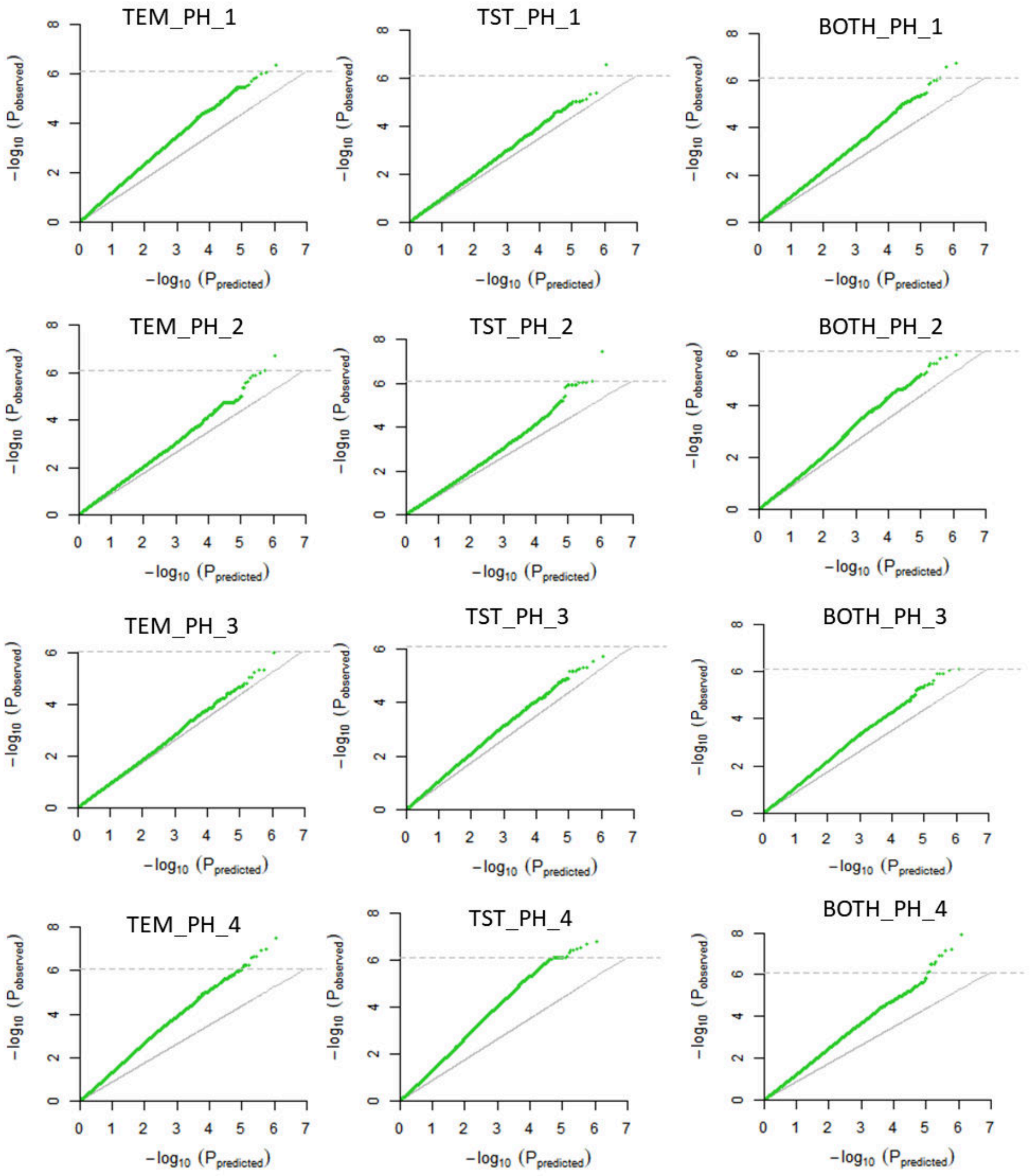

Figure S1. QQ plot of plant height at four stages among temperate (TEM), tropical (TST), and both (BOTH) maize groups.

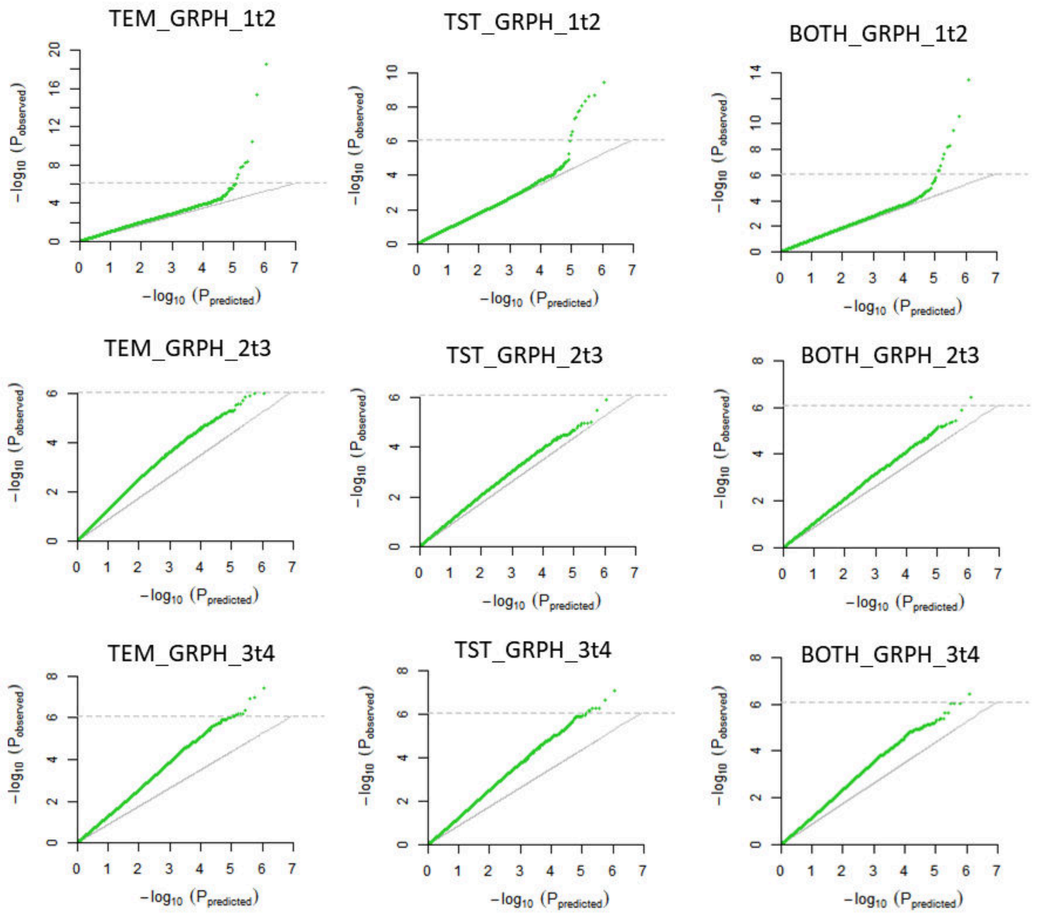

Figure S2. QQ plot for growth rate at three time intervals temperate (TEM), tropical (TST), and both (BOTH) maize groups.

Supplementary Table 1. QTLs for PH among the TEM, TST and BOTH groups.

Trait	Group	Chr	Start[a]	End[b]	Peak_SNP	P
PH_1	BOTH	2	197635457	197835457	chr2.S_197735457	7.86E-07
PH_1	BOTH	7	6027999	6227999	chr7.S_6127999	1.86E-07
PH_1	BOTH	7	153724681	153924801	chr7.S_153824801	2.58E-07
PH_1	TEM	1	166672606	166872606	chr1.S_166772606	8.71E-07
PH_1	TEM	9	3232261	3432838	chr9.S_3332261	4.18E-07
PH_1	TST	4	236312871	236512871	chr4.S_236412871	2.58E-07
PH_2	TEM	4	21483009	21683009	chr4.S_21583009	1.98E-07
PH_2	TEM	5	22503598	22703598	chr5.S_22603598	8.41E-07
PH_2	TST	2	174993293	175193293	chr2.S_175093293	7.87E-07
PH_2	TST	5	171606638	171806688	chr5.S_171706638	9.41E-07
PH_2	TST	7	91115527	91315527	chr7.S_91215527	9.40E-07
PH_2	TST	10	22538497	22738497	chr10.S_22638497	3.67E-08
PH_3	BOTH	2	204773427	204973427	chr2.S_204873427	7.93E-07
PH_3	BOTH	4	5609380	5809380	chr4.S_5709380	9.00E-07
PH_4	BOTH	2	235946078	236146986	chr2.S_236046078	3.09E-07
PH_4	BOTH	3	204183178	204383178	chr3.S_204283178	7.60E-07
PH_4	BOTH	3	205917134	206117134	chr3.S_206017134	3.00E-07
PH_4	BOTH	4	186932876	187134559	chr4.S_187032916	6.20E-08
PH_4	BOTH	6	110193972	110393972	chr6.S_110293972	2.50E-07
PH_4	BOTH	10	94153633	94353633	chr10.S_94253633	1.29E-08
PH_4	TEM	2	2493367	4362363	chr2.S_2593367	1.17E-07
PH_4	TEM	2	6528201	7289631	chr2.S_6628246	2.70E-08
PH_4	TEM	2	16119630	17890234	chr2.S_16219630	3.08E-08
PH_4	TEM	2	192332591	192532591	chr2.S_192432591	5.51E-07
PH_4	TEM	2	216911715	217111715	chr2.S_217011715	9.79E-08
PH_4	TEM	3	117491245	117691245	chr3.S_117591245	7.72E-07
PH_4	TEM	3	137582077	137782905	chr3.S_137682077	5.83E-07
PH_4	TEM	4	3653114	3860969	chr4.S_3753114	3.01E-07
PH_4	TEM	6	103048703	103249009	chr6.S_103148703	2.34E-07
PH_4	TST	1	13896351	14097341	chr1.S_13996351	2.11E-07
PH_4	TST	2	196906509	197109803	chr2.S_197008672	2.20E-07
PH_4	TST	3	205924534	206125361	chr3.S_206024534	9.03E-07
PH_4	TST	6	123101532	123350154	chr6.S_123201532	8.18E-07
PH_4	TST	8	77335430	79154142	chr8.S_78079487	2.08E-07
PH_4	TST	10	94153633	94353633	chr10.S_94253633	1.60E-07
PH_4	TST	10	113614123	113814123	chr10.S_113714123	9.65E-07

PH_4	TST	10	127912042	128211059	chr10.S_128027152	2.78E-08
PH_4	TST	10	131906681	132106681	chr10.S_132006681	3.88E-07

Note: a and b represent the position based on maize B73 RefGen_v2.

Supplementary Table 2. QTLs for GRPH among the TEM, TST and BOTH groups.

Trait	Group	Chr	Start[a]	End[b]	Peak_SNP	P
GRPH_1t2	BOTH	2	23216589	23416589	chr2.S_23316589	1.87E-07
GRPH_1t2	BOTH	2	212287814	212487814	chr2.S_212387814	3.51E-10
GRPH_1t2	BOTH	3	6398175	6598175	chr3.S_6498175	6.98E-09
GRPH_1t2	BOTH	3	70902400	71102400	chr3.S_71002400	2.25E-08
GRPH_1t2	BOTH	4	5062166	5262166	chr4.S_5162166	4.17E-07
GRPH_1t2	BOTH	4	240344524	240544524	chr4.S_240444524	3.84E-14
GRPH_1t2	BOTH	5	18530038	18730038	chr5.S_18630038	2.87E-11
GRPH_1t2	BOTH	5	212265400	212465400	chr5.S_212365400	4.87E-07
GRPH_1t2	BOTH	6	3134868	3334868	chr6.S_3234868	5.59E-08
GRPH_1t2	BOTH	6	133540862	133740862	chr6.S_133640862	5.60E-09
GRPH_1t2	TEM	1	75050818	75250818	chr1.S_75150818	9.24E-07
GRPH_1t2	TEM	2	223789903	223989903	chr2.S_223889903	1.84E-08
GRPH_1t2	TEM	4	185087603	185287603	chr4.S_185187603	9.84E-08
GRPH_1t2	TEM	4	240344524	240544524	chr4.S_240444524	2.93E-19
GRPH_1t2	TEM	5	14708300	14908300	chr5.S_14808300	2.24E-07
GRPH_1t2	TEM	5	203514344	203714344	chr5.S_203614344	1.49E-08
GRPH_1t2	TEM	5	211609549	211809549	chr5.S_211709549	4.72E-09
GRPH_1t2	TEM	7	97236473	97436473	chr7.S_97336473	3.62E-11
GRPH_1t2	TEM	7	136642692	136842692	chr7.S_136742692	5.23E-16
GRPH_1t2	TEM	10	110197924	110397924	chr10.S_110297924	6.66E-09
GRPH_1t2	TST	1	259423956	259623956	chr1.S_259523956	9.56E-07
GRPH_1t2	TST	2	23216589	23416589	chr2.S_23316589	2.53E-09
GRPH_1t2	TST	3	9712288	9912288	chr3.S_9812288	4.89E-08
GRPH_1t2	TST	3	185870856	186070856	chr3.S_185970856	3.61E-10
GRPH_1t2	TST	5	2261135	2461135	chr5.S_2361135	2.86E-07
GRPH_1t2	TST	6	35580970	35780970	chr6.S_35680970	1.99E-09
GRPH_1t2	TST	7	82282007	82482007	chr7.S_82382007	1.90E-08
GRPH_1t2	TST	7	100304245	100504245	chr7.S_100404245	4.37E-07

GRPH_1t2	TST	7	137355421	137555421	chr7.S_137455421	4.13E-08
GRPH_1t2	TST	9	25710016	25910016	chr9.S_25810016	8.97E-09
GRPH_1t2	TST	10	147605083	147805083	chr10.S_147705083	4.51E-09
GRPH_2t3	BOTH	2	191564333	191764333	chr2.S_191664333	3.85E-07
GRPH_3t4	BOTH	7	5512970	5713218	chr7.S_5612970	9.50E-07
GRPH_3t4	BOTH	7	171596629	171796629	chr7.S_171696629	2.64E-07
GRPH_3t4	TEM	2	2493367	4362363	chr2.S_2593367	1.17E-07
GRPH_3t4	TEM	2	16119630	17890234	chr2.S_16219630	3.08E-08
GRPH_3t4	TEM	3	50505440	50705440	chr3.S_50605440	2.17E-07
GRPH_3t4	TEM	6	74766193	74966193	chr6.S_74866193	6.39E-07
GRPH_3t4	TEM	7	101026820	101226820	chr7.S_101126820	7.26E-07
GRPH_3t4	TEM	7	138277897	138477897	chr7.S_138377897	4.04E-07
GRPH_3t4	TEM	7	140324272	140912196	chr7.S_140812196	8.34E-09
GRPH_3t4	TEM	7	143317492	143517492	chr7.S_143417492	9.05E-07
GRPH_3t4	TEM	8	8457746	8657746	chr8.S_8557746	7.76E-07
GRPH_3t4	TEM	8	13102391	13302391	chr8.S_13202391	2.66E-07
GRPH_3t4	TST	1	39038208	39238208	chr1.S_39138208	1.50E-07
GRPH_3t4	TST	7	125429820	125629820	chr7.S_125529820	6.70E-07
GRPH_3t4	TST	8	4924579	5124645	chr8.S_5024579	8.60E-08
GRPH_3t4	TST	8	77335430	79154142	chr8.S_78079487	2.08E-07
GRPH_3t4	TST	10	4637142	4837142	chr10.S_4737142	6.40E-07
GRPH_3t4	TST	10	115024110	115224110	chr10.S_115124110	8.16E-07

Note: a and b represent the position based on maize B73 RefGen_v2.

Supplementary Table 3. Unique QTLs for both PH and GRPH traits among the TEM, TST and BOTH groups.

Traits	Group	Chr	Start[a]	End[b]	Peak_SNP	P	Candidate Gene	Name	Annotation	Reference
PH_1	TEM	1	166672606	166872606	chr1.S_166772606	8.71E-07	GRMZM2G 124423	---	RING-H2 finger protein	11,12
PH_1	TST	4	236312871	236512871	chr4.S_236412871	2.58E-07	GRMZM2G 471304	*SAUR71*	Auxin responsive protein SAUR71	9
PH_1	TEM	9	3232261	3432838	chr9.S_3332261	4.18E-07	GRMZM2G 136831	---	Serine/threonine protein kinase WNK (With No Lysine)-related	This study
PH_1, GRPH_3t4	BOTH	7	5512970	6227999	chr7.S_6127999	1.86E-07	GRMZM2G 090104	---	Galactose oxidase/kelch repeat superfamily protein	12
PH_2	TST	2	174993293	175193293	chr2.S_175093293	7.87E-07	GRMZM2G 100484	---	Nucleobase ascorbate transporter 2	This study
PH_2	TEM	4	21483009	21683009	chr4.S_21583009	1.98E-07	GRMZM2G 015854	---	---	This study
PH_2	TEM	5	22503598	22703598	chr5.S_22603598	8.41E-07	GRMZM2G 002859	---	SH3 domain containing protein	11

PH_2	TST	5	171606638	171806688	chr5.S_171706638	9.41E-07	GRMZM2G 000278	*spk1*	Stress-induced protein kinase1	42
PH_2	TST	7	91115527	91315527	chr7.S_91215527	9.40E-07	GRMZM2G 519152	---	---	This study
PH_2	TST	10	22538497	22738497	chr10.S_22638497	3.67E-08	---	---	---	This study
PH_3	BOTH	2	204773427	204973427	chr2.S_204873427	7.93E-07	GRMZM2G 067520	---	Protein kinase superfamily protein	11
PH_4	TST	1	13896351	14097341	chr1.S_13996351	2.11E-07	GRMZM2G 123986	---	Pollenless3	This study
PH_4	TEM	2	6528201	7289631	chr2.S_6628246	2.70E-08	GRMZM5G 899080	*propep1*	Precursor elicitor peptide1	This study
PH_4	TEM	2	216911715	217111715	chr2.S_217011715	9.79E-08	GRMZM2G 172621	---	B3 domain containing transcription repressor VAL2	This study
PH_4	BOTH	2	235946078	236146986	chr2.S_236046078	3.09E-07	GRMZM2G 070542	---	T-complex protein 1 subunit epsilon	This study
PH_4	TEM	3	117491245	117691245	chr3.S_117591245	7.72E-07	GRMZM2G 441768	---	F-box/LRR repeat protein 17	This study
PH_4	TEM	3	137582077	137782905	chr3.S_137682077	5.83E-07	GRMZM2G 422210	---	Cox19 family protein (CHCH motif)	This study

PH_4	TST,BOTH	3	204183178	206125361	chr3.S_204283178	7.60E-07	GRMZM2G 108424	*SMH4*	Single myb histone 4	12
PH_4	BOTH	4	186932876	187134559	chr4.S_187032916	6.20E-08	GRMZM2G 140047	---	PP2A regulatory subunit TAP46	This study
PH_4	TEM	6	103048703	103249009	chr6.S_103148703	2.34E-07	GRMZM2G 079080	---	Putative argonaute family protein	42
PH_4	BOTH	6	110193972	110393972	chr6.S_110293972	2.50E-07	GRMZM2G 038691	*POB1*	BTB/POZ domain containing protein POB1	42
PH_4	TST	6	123101532	123350154	chr6.S_123201532	8.18E-07	GRMZM2G 426175	---	Flavanone 3-dioxygenase	This study
PH_4	TST,BOTH	10	94153633	94353633	chr10.S_94253633	1.60E-07	GRMZM2G 381691	*ZmCCT*	CO CO-LIKE TIMING OF CAB1 protein domain1	43
PH_4	TST	10	113614123	113814123	chr10.S_113714123	9.65E-07	GRMZM2G 147424	*sfb1*	SF1 binding protein candidate1	This study
PH_4	TST	10	127912042	128211059	chr10.S_128027152	2.78E-08	GRMZM2G 149994	---	Cell division cycle 48B	This study
PH_4	TST	10	131906681	132106681	chr10.S_132006681	3.88E-07	GRMZM2G 320135	---	ATP-dependent Clp protease proteolytic subunit	9

PH_4, GRPH_3t4	TEM	2	2493367	4362363	chr2.S_2593367	1.17E-07	GRMZM2G 034840	*ARFTF4*	Auxin response factor 4	9
PH_4, GRPH_3t4	TEM	2	16119630	17890234	chr2.S_16219630	3.08E-08	GRMZM2G 142664	---	Auxin-independent growth promoter	This study
PH_4, GRPH_3t4	TST	8	77335430	79154142	chr8.S_78079487	2.08E-07	GRMZM2G 126026	---	CCT motif family protein	This study
PH_4,PH_1	TST,BOTH	2	196906509	197835457	chr2.S_197008672	2.20E-07	GRMZM2G 042712	*SAUR61*	SAUR44-auxin-respo nsive SAUR family member	11
GRPH_1t2	TEM	1	75050818	75250818	chr1.S_75150818	9.24E-07	GRMZM2G 042881	---	Eukaryotic protein of unknown function (DUF872)	This study
GRPH_1t2	TST	1	259423956	259623956	chr1.S_259523956	9.56E-07	GRMZM2G 092433	---	Amino acid transmembrane transporter activity	This study
GRPH_1t2	TST,BOTH	2	23216589	23416589	chr2.S_23316589	2.53E-09	GRMZM2G 136486	---	Elicitor responsive protein 3	This study
GRPH_1t2	BOTH	2	212287814	212487814	chr2.S_212387814	3.51E-10	AC194203. 3_FG003	---	Lipid transport	11,12
GRPH_1t2	TEM	2	223789903	223989903	chr2.S_223889903	1.84E-08	GRMZM2G 003043	*RBR1*	Retinoblastoma related protein 1	This study

GRPH_1t2	BOTH	3	6398175	6598175	chr3.S_6498175	6.98E-09	GRMZM2G 115834	---	Signal transduction	This study
GRPH_1t2	TST	3	9712288	9912288	chr3.S_9812288	4.89E-08	GRMZM2G 421604	---	Major facilitator superfamily protein	This study
GRPH_1t2	BOTH	3	70902400	71102400	chr3.S_71002400	2.25E-08	GRMZM5G 833328	---	---	12
GRPH_1t2	TST	3	185870856	186070856	chr3.S_185970856	3.61E-10	GRMZM2G 021589	---	TRAM, LAG1 and CLN8 (TLC) lipid-sensing domain containing protein	This study
GRPH_1t2	TEM	4	185087603	185287603	chr4.S_185187603	9.84E-08	GRMZM2G 020864	---	Pentatricopeptide repeat protein PPR868-14 isoform 1	This study
GRPH_1t2	TEM, BOTH	4	240344524	240544524	chr4.S_240444524	2.93E-19	GRMZM2G 384293	*emb14*	Embryo defective 14	9
GRPH_1t2	TST	5	2261135	2461135	chr5.S_2361135	2.86E-07	GRMZM2G 144188	---	Dof zinc finger protein DOF3.6	This study
GRPH_1t2	TEM	5	14708300	14908300	chr5.S_14808300	2.24E-07	GRMZM2G 066191	*TUBB4*	Tubulin beta-4 chain	This study
GRPH_1t2	BOTH	5	18530038	18730038	chr5.S_18630038	2.87E-11	GRMZM2G 048363	---	Inorganic phosphate cotransporter	This study

GRPH_1t2	TEM	5	203514344	203714344	chr5.S_203614344	1.49E-08	GRMZM5G 837108	*ant1*	Adenine nucleotide translocator1	11,12, 13,42
GRPH_1t2	TEM, BOTH	5	211609549	212465400	chr5.S_211709549	4.72E-09	GRMZM5G 893117	*GRF9*	Growth-regulating factor 6	9,12
GRPH_1t2	BOTH	6	3134868	3334868	chr6.S_3234868	5.59E-08	GRMZM2G 173962	---	Protein kinase superfamily protein	This study
GRPH_1t2	TST	6	35580970	35780970	chr6.S_35680970	1.99E-09	GRMZM2G 448466	---	Mannose 6 phosphate isomerase	This study
GRPH_1t2	BOTH	6	133540862	133740862	chr6.S_133640862	5.60E-09	GRMZM2G 052412	---	Transcribed locus	This study
GRPH_1t2	TST	7	82282007	82482007	chr7.S_82382007	1.90E-08	GRMZM2G 106950	---	Indole-3-glycerol-pho sphate synthase	This study
GRPH_1t2	TEM	7	97236473	97436473	chr7.S_97336473	3.62E-11	GRMZM2G 132184	---	Protein kinase superfamily protein	This study
GRPH_1t2	TST	7	100304245	100504245	chr7.S_100404245	4.37E-07	GRMZM2G 126408	---	---	This study
GRPH_1t2	TST	9	25710016	25910016	chr9.S_25810016	8.97E-09	GRMZM2G 119411	---	Zn-dependent exopeptidases superfamily protein	This study

GRPH_1t2	TEM	10	110197924	110397924	chr10.S_110297924	6.66E-09	GRMZM2G 153215	---	ATGP4; Uncharacterized protein	This study
GRPH_1t2	TST	10	147605083	147805083	chr10.S_147705083	4.51E-09	GRMZM2G 475882	*ARFTF3 0*	Auxin response factor 8	This study
GRPH_1t2, GRPH_3t4	TEM,TST	7	136642692	140912196	chr7.S_140812196	8.34E-09	GRMZM2G 083102	---	Ribulose phosphate 3-epimerase	12
GRPH_1t2, PH_3,PH_4	TEM	4	3653114	5809380	chr4.S_3753114	3.01E-07	GRMZM2G 165383	---	3-phosphoserine phosphatase	11
GRPH_2t3, PH_4	BOTH, TEM	2	191564333	192532591	chr2.S_191664333	3.85E-07	GRMZM2G 040911	*pin11*	Putative auxin efflux carrier PIN5c	This study
GRPH_3t4	TST	1	39038208	39238208	chr1.S_39138208	1.50E-07	GRMZM2G 160237	---	Serine/threonine protein phosphatase	12
GRPH_3t4	TEM	3	50505440	50705440	chr3.S_50605440	2.17E-07	GRMZM2G 123765	---	Copper exporting ATPase	This study
GRPH_3t4	TEM	6	74766193	74966193	chr6.S_74866193	6.39E-07	AC186592. 3_FG003	---	---	This study
GRPH_3t4	TEM	7	101026820	101226820	chr7.S_101126820	7.26E-07	GRMZM2G 457407	---	---	This study
GRPH_3t4	TST	7	125429820	125629820	chr7.S_125529820	6.70E-07	GRMZM2G 136734	---	Lipid metabolic process	This study

GRPH_3t4	TST	8	4924579	5124645	chr8.S_5024579	8.60E-08	GRMZM2G 475017	*c3h23*	C3H transcription factor 323	12
GRPH_3t4	TEM	8	8457746	8657746	chr8.S_8557746	7.76E-07	GRMZM2G 396541	---	Sulfated surface glycoprotein 185	This study
GRPH_3t4	TEM	8	13102391	13302391	chr8.S_13202391	2.66E-07	GRMZM2G 006497	*Wrky49*	Putative WRKY DNA-binding domain superfamily protein	12
GRPH_3t4	TST	10	4637142	4837142	chr10.S_4737142	6.40E-07	GRMZM2G 003732	*U3*	U3 small nucleolar RNA-associated protein 25	42
GRPH_3t4	TST	10	115024110	115224110	chr10.S_115124110	8.16E-07	GRMZM2G 060720	---	VQ motif family protein	This study

Note: a and b represent the position based on maize B73 RefGen_v2.

References

42. Weng, J. *et al*. Genome-wide association study identifies candidate genes that affect plant height in Chinese elite maize (*Zea mays* L.) inbred lines. *PLoS One* 6, e29229 (2011).

43. Hung, H. Y. *et al*. *ZmCCT* and the genetic basis of day-length adaptation underlying the postdomestication spread of maize. *Proceedings of the National Academy of Sciences of the United States of America* 109, 1913–1921 (2012).
